# GmRPS5 Promoter‐Driven CRISPR/LbCas12a Efficiently Generates Soybean Sextuple Mutants

**DOI:** 10.1111/pbi.70588

**Published:** 2026-02-05

**Authors:** Xiangchao Kong, Kexin Fan, Cuiping Xin, Minhui Lu, Yunlu Shi, Jie Jin, Qi‐Jun Chen

**Affiliations:** ^1^ State Key Laboratory of Plant Environmental Resilience, College of Biological Sciences China Agricultural University Beijing China; ^2^ Center for Crop Functional Genomics and Molecular Breeding China Agricultural University Beijing China; ^3^ Biorun Biosciences Co., LTD Wuhan China

CRISPR/SpCas9 remains the predominant genome editing platform in soybean (Freitas‐Alves et al. 2025). Among stable soybean transformants, the CaMV 35S and 2×CaMV 35S promoters are the most frequently used, accounting for 33 of 70 studies surveyed (Freitas‐Alves et al. 2025). The remaining 37 studies employed alternative promoters, including GmUbi3, GmScreamM4, AtUBQ10, AtRPS5A (ribosomal protein S5A), an AtEC1.2‐AtEC1.1 fusion promoter, ZmUbi, PcUbi and DaMV 35S (Freitas‐Alves et al. 2025). The soybean GmEF1A2 gene, which encodes elongation factor 1‐alpha (EF1A), is constitutively and highly expressed across multiple tissues; its promoter (GmScreamM4) has supported efficient CRISPR/Cas9 genome editing (Bai et al. 2020).

Genome editing mediated by LbCas12a and Cas12i3 has recently been reported in transgenic soybean plants and hairy roots (Freitas‐Alves et al. 2025; Gao et al. 2024; Valentine et al. 2024; Xie et al. 2024; Zhong et al. 2024). In these studies, the eFMV‐AtEF1A, Medicago ubiquitin (MtUbq2), CaMV 35S or GmUbi promoters were used to drive LbCas12a, including variants harbouring D156R or C965S substitutions; by contrast, the GmEF1A2 (GmScreamM4) promoter was used to drive a Cas12i3 variant.

We previously reported that the LbCas12a variant ttLbCas12a Ultra V2 (ttLbUV2), which harbours the D156R and E795L mutations derived from low‐temperature‐tolerant and highly active variants, respectively, and incorporates optimised nuclear localisation signal (NLS) sequences, exhibits high efficiency for multiplex genome editing in Arabidopsis (Xin et al. 2025). Given that RPS5 promoters effectively drive LbCas12a‐based editors in Arabidopsis (Xin et al. 2025), prime editors in tomato (Lu et al. 2021) and base editors in *Salvia miltiorrhiza* (Danshen) (Yao et al. 2025), we hypothesised that expressing ttLbUV2 under the control of a soybean RPS5 promoter would similarly enable high‐efficiency multiplex genome editing in soybean. Here, we show that, among four soybean RPS5 promoters evaluated, GmRPS5‐9 most effectively drove an LbCas12a variant bearing D156R and E795L substitutions with optimised NLSs, enabling efficient generation of sextuple mutants in soybean.

Four RPS5 promoters were cloned from the soybean cultivar Tianlong #1 and designated RPS5‐2, RPS5‐9, RPS5‐12 and RPS5‐13 based on chromosomal location. As controls, two additional promoters—GmScreamM4 and GmScreamM8, which are associated with EF1A‐encoding genes and exhibit high expression (Bai et al. 2020)—were also cloned. Each of the six promoters was used to drive ttLbUV2, whereas the AtU6‐26 and GmU6 promoters transcribed four crRNAs targeting six genes (Figure 1a).

**FIGURE 1:**
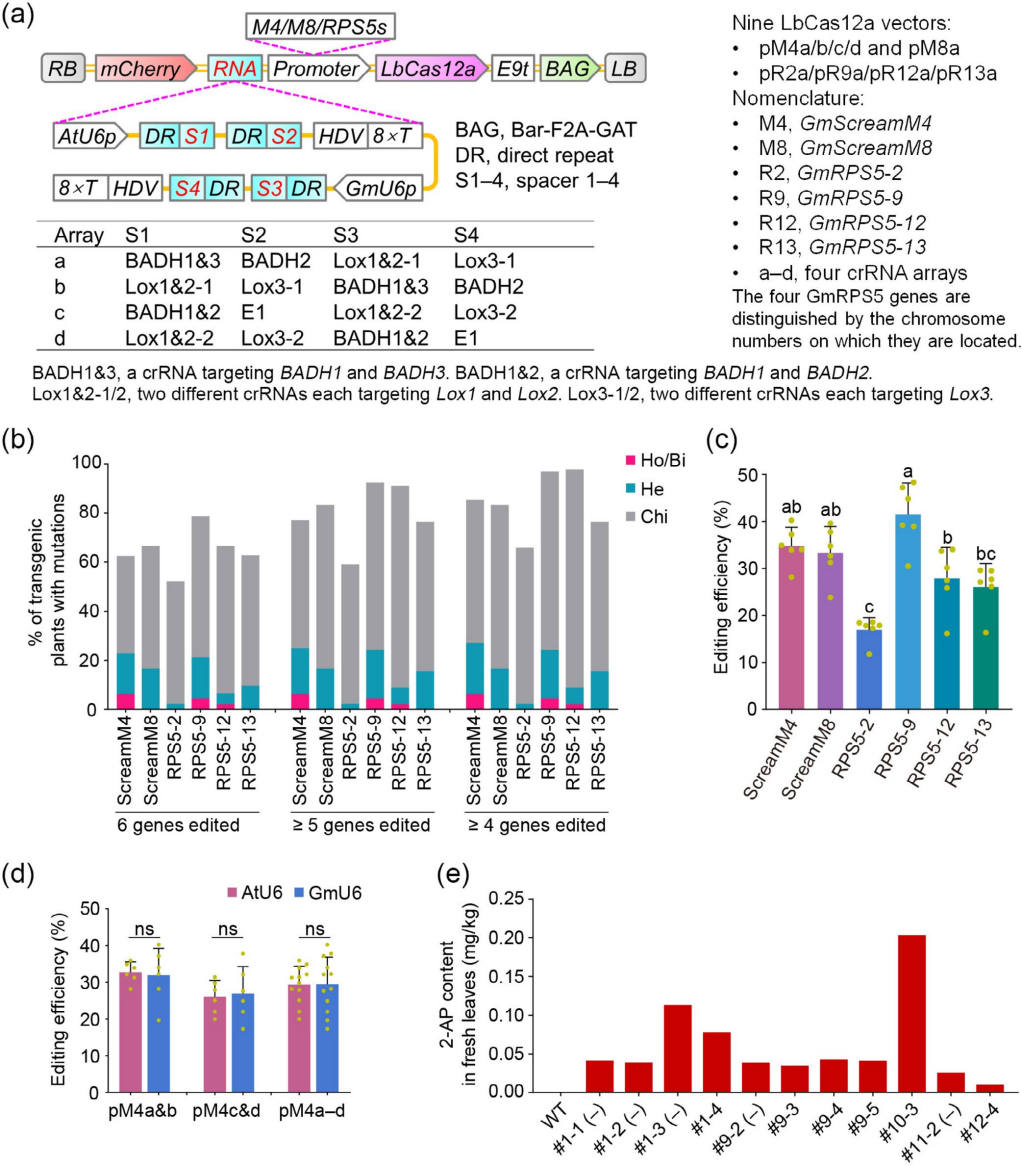
GmRPS5‐9 promoter‐driven LbCas12a enables efficient sextuple mutagenesis in soybean. (a) T‐DNA architectures of the binary vectors. (b) Sorting‐based editing efficiencies for six promoters across six targets in 272 transgenic lines. Ho/Bi, homozygous or biallelic; He, heterozygous; Chi, chimeric. (c) Mean editing efficiency of each promoter across six targets (mean ± SD; *n* = 6 targets). One‐way ANOVA with Tukey's multiple‐comparisons test; bars sharing a lowercase letter are not significantly different (*p* > 0.05). (d) Comparison of AtU6 and GmU6 across six targets (pM4a + pM4b), six targets (pM4c + pM4d), and all 12 targets combined. Two‐tailed Student's *t*‐test; ns, not significant (*p* > 0.05). (e) The 2‐AP content of 11 homozygous or biallelic sextuple mutants. The minus sign in parentheses denotes the absence of T‐DNA.

To assess multiplex editing, we selected two betaine aldehyde dehydrogenase (BADH) genes, three lipoxygenase genes (*Lox1*, *Lox2* and *Lox3*), and the major maturity gene *E1*. Disruption of *BADH1* and *BADH2* confers strong aroma in soybean (Xie et al. 2024); mutations in the three lipoxygenase genes reduce beany flavour (Wang et al. 2020); and targeted mutation of *E1* decreases photoperiod sensitivity (Gao et al. 2024). Four crRNA arrays (a–d) were designed, each comprising four crRNAs, to target these loci (Figure 1a). Arrays a and b share identical crRNAs but differ in the Pol III promoter used for crRNA transcription (AtU6 vs. GmU6), as do arrays c and d (Figure 1a). In arrays a and b, we adopted two previously reported Cas12i3 spacers targeting *BADH1* and *BADH2* (Xie et al. 2024). The crRNA targeting *BADH1* also matches *Glyma.11G164664*, a putative BADH pseudogene herein provisionally designated *BADH3*. Accordingly, this crRNA was named BADH1&3 (Figure 1a). In arrays c and d, the BADH1&2 crRNA perfectly matches the *BADH1* site but carries a PAM‐distal mismatch relative to *BADH2*. Nine LbCas12a constructs were assembled by combining the six promoters with the four crRNA arrays (Figure 1a). We obtained 48, 46, 34, 33, 18, 44, 66, 45 and 51 transgenic lines for pM4a–d, pM8a, pR2a, pR9a, pR12a and pR13a, respectively (M denotes ScreamM; R denotes RPS5; a–d denote crRNA arrays).

Mutations in each transgenic line were assessed by sorting‐based and reads‐based analyses (Figure 1b, Figure S1, Tables S1–S6). All six promoters effectively drove LbCas12a, with GmRPS5‐9 and GmScreamM4 yielding the highest efficiencies (Figure 1b,c; Figure S1a,b, Tables S3 and S4). The frequencies of homozygous and heterozygous sextuple mutants were 21.2% (14/66) for pR9a (GmRPS5‐9) and 22.9% (11/48) for pM4a (GmScreamM4); the frequencies of chimeric mutations across the six genes were 57.6% (38/66) and 39.6% (19/48), respectively (Figure 1b, Table S3). Sextuple genotypes predominated over other higher‐order classes (e.g., quintuple or quadruple), suggesting low target bias (Figure 1b, Table S3). Reads‐based analysis likewise indicated that GmRPS5‐9 slightly outperformed GmScreamM4 and efficiently supported CRISPR/LbCas12a for multiplex genome editing in soybean (Figure 1c, Figure S1c, Table S4). We detected no significant difference in editing efficiency between GmU6 and AtU6 across the six targets in pM4a/pM4b, across the six targets in pM4c/pM4d, or all 12 targets combined (Figure 1a,d, Figure S1a,c,d, Tables S5–S7). Thus, AtU6‐26, like GmU6, supports efficient genome editing in soybean.

We identified T‐DNA‐free T1 plants that were either homozygous or biallelic sextuple mutants, derived from T0 homozygous (pR9a #1) or heterozygous (pR9a #11) mutant lines (Table S8). These results demonstrate that the mutations in the six genes in T0 plants were stably transmitted to T1 plants. Interestingly, a T0 line (pR9a #22) carrying chimeric mutations in three genes and heterozygous mutations in the remaining three genes gave rise to T1 plants that were homozygous or biallelic sextuple mutants. We quantified 2‐acetyl‐1‐pyrroline (2‐AP) content in the leaves of 11 homozygous or biallelic sextuple mutants, and all exhibited increased 2‐AP levels compared with the wild type (Figure 1e, Table S8).

In summary, expression of an LbCas12a variant bearing D156R and E795L substitutions and optimised NLSs under the GmRPS5‐9 promoter enables efficient multiplex genome editing in soybean.

## Author Contributions

Q.‐J.C. conceived and designed the research. X.K., K.F., C.X., M.L., Y.S. and J.J. conducted the experiments and analysed the data. Q.‐J.C., X.K. and K.F. wrote the manuscript.

## Conflicts of Interest

The authors declare no conflicts of interest.

## Supporting information


**Data S1:** pbi70588‐sup‐0001‐DataS1.zip.

## Data Availability

The data that supports the findings of this study are available in the main text and Supporting Information.
