# Supplementary material for: GmRPS5 Promoter‐Driven CRISPR/LbCas12a Efficiently Generates Soybean Sextuple Mutants
Source: Plant Biotechnol J. 2026 Feb 5;24(5):3494–6. doi: 10.1111/pbi.70588 (PMC13110138; doi:10.1111/pbi.70588)
Supplement: Supplementary file 1 — Data S1: pbi70588‐sup‐0001‐DataS1.zip. [file PBI-24-3494-s001.zip › pbi70588-sup-0001-DataS1/pbi70588-sup-0001-Supinfo.pdf]

Supporting information

GmRPS5 Promoter-Driven CRISPR/LbCas12a Efficiently Generates Soybean Sextuple Mutants

Xiangchao **Kong**[1,†], Kexin **Fan**[1,†], Cuiping **Xin**[1], Minhui **Lu**[2], Yunlu **Shi**[2], Jie **Jin**[3], Qi-Jun **Chen**[1,2*]

[1]State Key Laboratory of Plant Environmental Resilience, College of Biological Sciences, China Agricultural University, Beijing 100193, China

[2]Center for Crop Functional Genomics and Molecular Breeding, China Agricultural University, Beijing 100193, China

[3]Biorun Biosciences Co., LTD, Wuhan 430072, China

[†]These authors contributed equally to this article.

[*]Correspondence: Qi-Jun Chen (qjchen@cau.edu.cn)

Table of contents

Figure S1. Editing efficiencies of nine LbCas12a vectors across 12 targets 3
Table S1. Mutations in the six genes // the crRNA arrays a or b 5
Table S2. Mutations in the six genes // the crRNA arrays c or d 6
Table S3. Sorting-based editing efficiencies // the crRNA arrays a or b 7
Table S4. Reads-based editing efficiencies of the crRNA arrays a and b 8
Table S5. Sorting-based editing efficiencies // the crRNA arrays c or d 9
Table S6. Reads-based editing efficiencies of the crRNA arrays c and d 10
Table S7. Comparison of two promoters of crRNAs 11
Table S8. Analysis of mutations and the content of 2-AP 12
Table S9. Primer sequences used in this study 13
Table S10. Sequences of target sites 15
Sequences S1. Sequences of the six promoters and crRNA cassettes.. 16
Methods S1. Vector construction, mutation analysis, and related procedures.. 21

Figure S1. Editing efficiencies of nine LbCas12a vectors across 12 targets

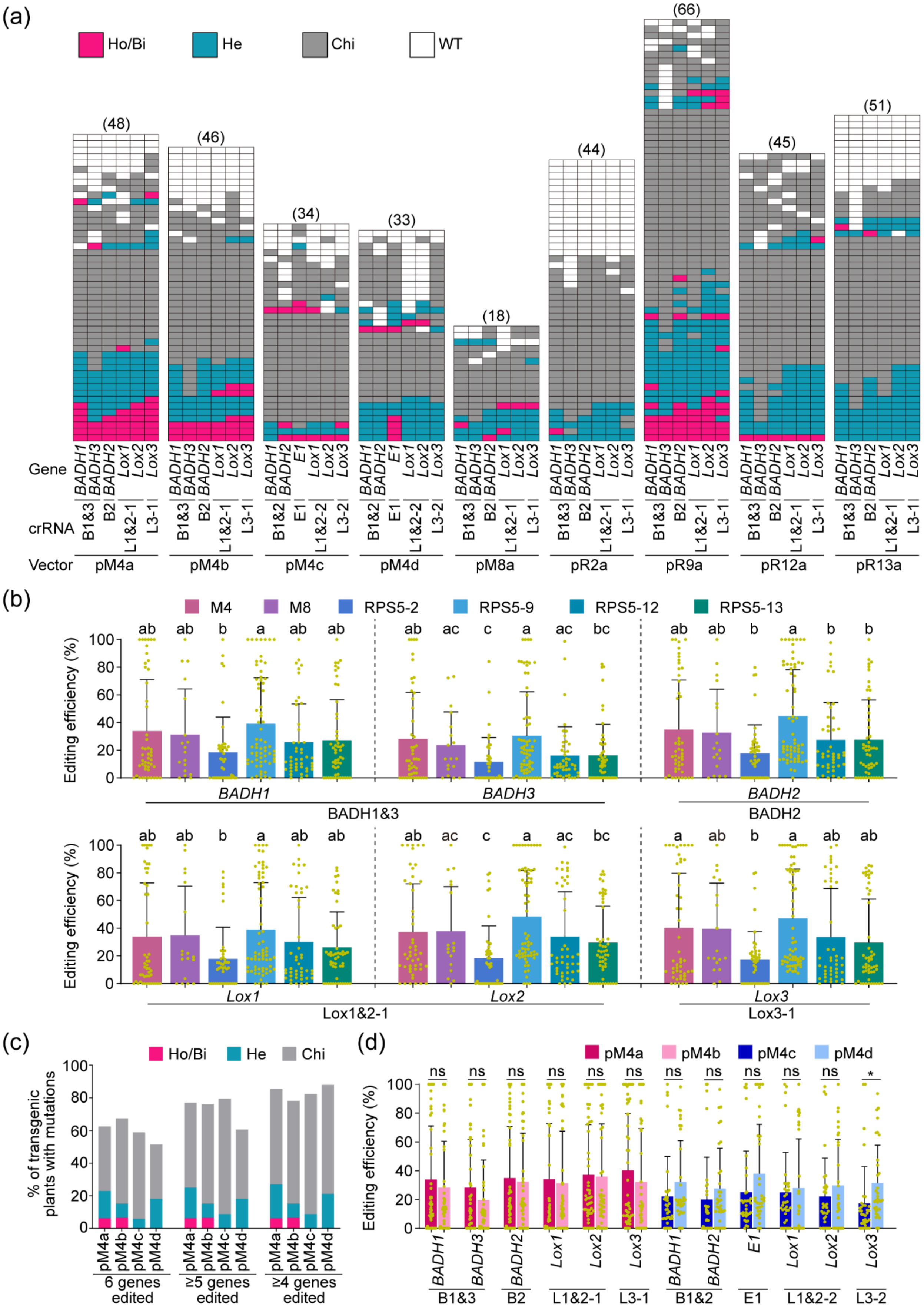

Figure S1 Editing efficiencies of nine LbCas12a vectors across 12 targets. (a) Summary of mutation types across six genes for each transgenic line. The numbers denote the counts of transgenic lines. Ho/Bi, homozygous or biallelic; He, heterozygous; Chi, chimeric. (b) Reads-based editing efficiencies for the six promoters across the six targets in the 272 lines. Columns and error bars represent the mean and SD, respectively. One-way ANOVA followed by Tukey's multiple-comparisons test was used; bars sharing a lowercase letter indicate no significant difference ($P > 0.05$). (c) Sorting-based editing efficiencies for the four vectors yielding sextuple, quintuple, and quadruple mutants. (d) Reads-based editing efficiencies for the same four vectors across six targets. For each line, the editing efficiency at a given target equals the percentage of high-throughput sequencing (HTS) reads carrying mutations; the mean of these percentages across all lines harboring the same LbCas12a vector is reported as that vector's editing efficiency.

Table S1. Mutations in the six genes // the crRNA arrays a or b

Table S1. Mutations in the six genes in each line harboring the crRNA arrays a or b
(See the XLSX spreadsheet)

Table S2. Mutations in the six genes // the crRNA arrays c or d

Table S2. Mutations in the six genes in each line harboring the crRNA arrays c or d
(See the XLSX spreadsheet)

Table S3. Sorting-based editing efficiencies // the crRNA arrays a or b

Table S3. Sorting-based editing efficiencies for generation of sextuple, quintuple, and quadruple mutants harboring the crRNA arrays a or b

Number of edited genes	Vector	Ho/Bi	He	Chi	Total
6	pM4a	6.3% (3/48)	16.7% (8/48)	39.6% (19/48)	62.5% (30/48)
	pM8a	0.0% (0/18)	16.7% (3/18)	50.0% (9/18)	66.7% (12/18)
	pR2a	0.0% (0/44)	2.3% (1/44)	50.0% (22/44)	52.3% (23/44)
	pR9a	4.5% (3/66)	16.7% (11/66)	57.6% (38/66)	78.8% (52/66)
	pR12a	2.2% (1/45)	4.4% (2/45)	60.0% (27/45)	66.7% (30/45)
	pR13a	0.0% (0/51)	9.8% (5/51)	52.9% (27/51)	62.7% (32/51)
	pM4b	6.5% (3/46)	8.7% (4/46)	52.2% (24/46)	67.4% (31/46)
≥5	pM4a	6.3% (3/48)	18.8% (9/48)	52.1% (25/48)	77.1% (37/48)
	pM8a	0.0% (0/18)	16.7% (3/18)	66.7% (12/18)	83.3% (15/18)
	pR2a	0.0% (0/44)	2.3% (1/44)	56.8% (25/44)	59.1% (26/44)
	pR9a	4.5% (3/66)	19.7% (13/66)	68.2% (45/66)	92.4% (61/66)
	pR12a	2.2% (1/45)	6.7% (3/45)	82.2% (37/45)	91.1% (41/45)
	pR13a	0.0% (0/51)	15.7% (8/51)	60.8% (31/51)	76.5% (39/51)
	pM4b	6.5% (3/46)	8.7% (4/46)	60.9% (28/46)	76.1% (35/46)
≥4	pM4a	6.3% (3/48)	20.8% (10/48)	58.3% (28/48)	85.4% (41/48)
	pM8a	0.0% (0/18)	16.7% (3/18)	66.7% (12/18)	83.3% (15/18)
	pR2a	0.0% (0/44)	2.3% (1/44)	63.6% (28/44)	65.9% (29/44)
	pR9a	4.5% (3/66)	19.7% (13/66)	72.7% (48/66)	97.0% (64/66)
	pR12a	2.2% (1/45)	6.7% (3/45)	88.9% (40/45)	97.8% (44/45)
	pR13a	0.0% (0/51)	15.7% (8/51)	60.8% (31/51)	76.5% (39/51)
	pM4b	6.5% (3/46)	8.7% (4/46)	63.0% (29/46)	78.3% (36/46)

Ho/Bi: homozygous/biallelic mutants; He: heterozygous mutants; Chi: chimeric mutants. Homozygous/biallelic mutants denote that all targeted genes harbor homozygous/biallelic mutations. Heterozygous mutants have at least one gene with heterozygous mutations and the remaining genes with either heterozygous or homozygous mutations. Chimeric mutants have at least one gene with chimeric mutations and the other genes with different mutation types. The ratio of mutants to the total number of transgenic lines is indicated in parentheses.

Table S4. Reads-based editing efficiencies of the crRNA arrays a and b

Table S4. Reads-based editing efficiencies of the crRNA arrays a and b

Vector	No. of lines	*BADH1*	*BADH3*	*BADH2*	*Lox1*	*Lox2*	*Lox3*	Average	SD
pM4a	48	34.0%	28.2%	35.0%	34.0%	37.3%	40.3%	34.8%	4.0%
pM8a	18	31.3%	23.9%	32.7%	34.8%	37.8%	39.6%	33.4%	5.6%
pR2a	44	18.6%	11.8%	17.8%	17.9%	18.5%	17.3%	17.0%	2.6%
pR9a	66	39.2%	30.5%	44.9%	39.0%	48.3%	47.3%	41.5%	6.7%
pR12a	45	25.9%	16.2%	27.5%	30.2%	34.1%	33.7%	27.9%	6.6%
pR13a	51	27.1%	16.4%	27.7%	26.2%	29.5%	29.7%	26.1%	5.0%
pM4b	46	28.3%	19.6%	32.3%	31.3%	36.0%	32.2%	29.9%	5.6%

Table S5. Sorting-based editing efficiencies // the crRNA arrays c or d

Table S5. Sorting-based editing efficiencies for generation of sextuple, quintuple, and quadruple mutants harboring the crRNA arrays c or d

Number of edited genes	Vector	Ho/Bi	He	Chi	Total
6	pM4c	0.0% (0/34)	5.9% (2/34)	52.9% (18/34)	58.8% (20/34)
	pM4d	0.0% (0/33)	18.2% (6/33)	33.3% (11/33)	51.5% (17/33)
≥5	pM4c	0.0% (0/34)	8.8% (3/34)	70.6% (24/34)	79.4% (27/34)
	pM4d	0.0% (0/33)	18.2% (6/33)	42.4% (14/33)	60.6% (20/33)
≥4	pM4c	0.0% (0/34)	8.8% (3/34)	73.5% (25/34)	82.4% (28/34)
	pM4d	0.0% (0/33)	21.2% (7/33)	66.7% (22/33)	87.9% (29/33)

Ho/Bi: homozygous/biallelic mutants; He: heterozygous mutants; Chi: chimeric mutants. Homozygous/biallelic mutants denote that all targeted genes harbor homozygous/biallelic mutations. Heterozygous mutants have at least one gene with heterozygous mutations and the remaining genes with either heterozygous or homozygous mutations. Chimeric mutants have at least one gene with chimeric mutations and the other genes with different mutation types. The ratio of mutants to total number of transgenic lines is indicated in parentheses.

Table S6. Reads-based editing efficiencies of the crRNA arrays c and d

Table S6. Reads-based editing efficiencies of the crRNA arrays c and d

Vector	No. of lines	*BADH1*	*BADH2*	*E1*	*Lox1*	*Lox2*	*Lox3*	Average	SD
pM4c	34	22.0%	20.0%	25.1%	25.0%	22.1%	17.3%	21.9%	3.0%
pM4d	33	32.2%	27.7%	37.8%	28.0%	29.8%	31.5%	31.1%	3.7%

Table S7. Comparison of two promoters of crRNAs

Table S7. Comparison of two promoters of crRNAs

U6	Vector	Gene	Editing efficiency	SD
AtU6	pM4a	*BADH1*	34.0%	/
		BADH3	28.2%	/
		BADH2	35.0%	/
	pM4b	*Lox1*	31.3%	/
		Lox2	36.0%	/
		Lox3	32.2%	/
	pM4c	*BADH1*	22.0%	/
		BADH2	20.0%	/
		E1	25.1%	/
	pM4d	*Lox1*	28.0%	/
		Lox2	29.8%	/
		Lox3	31.5%	/
	Average (a&b)	/	32.8%	2.8%
	Average (c&d)	/	26.1%	4.5%
	Average (a–d)	/	29.4%	5.0%
GmU6	pM4b	*BADH1*	28.3%	/
		BADH3	19.6%	/
		BADH2	32.3%	/
	pM4a	*Lox1*	34.0%	/
		Lox2	37.3%	/
		Lox3	40.3%	/
	pM4d	*BADH1*	32.2%	/
		BADH2	27.7%	/
		E1	37.8%	/
	pM4c	*Lox1*	25.0%	/
		Lox2	22.1%	/
		Lox3	17.3%	/
	Average (a&b)	/	32.0%	7.3%
	Average (c&d)	/	27.0%	7.3%
	Average (a–d)	/	29.5%	7.4%

Table S8. Analysis of mutations and the content of 2-AP

Table S8. Analysis of mutations and the content of 2-AP in T1 plants from 8 T0 plants

(See the XLSX spreadsheet)

Table S9. Primer sequences used in this study

Table S9. Primer sequences used in this study

Name	Sequence	Purpose
M4-SpeF	ACATTACTAGTATTCCTGAACTTGTATTATTCAGTAGAT	Promoter cloning
M4-mHiR	ATGGTCGCATACATAGTAAAAGATTAAGGTTGATAACATGTGAACAAACCCACAA	
M4-mHiF	TTGTGGGTTTGTTCACATGTTATCAACCTTAATCTTTTACTATGTATGCGACCAT	
M4-XbaR	ATACCACGTCTAGATTACTTAAACTAGAATCAATTCAA	
M8-SpeF	ATTGTTGACTAGTATCCTTTTACGTGTGCTGTGAGACATTATC	
M8-XbaR	ACATTGGTCTAGATTCCTTAAATCTGCAAAAATCCAGAA	
R2-SpeF	ACATTATTACTAGTGCTTGAAGGGGCACCCACATTT	
R2-XbaR	AACCAACTCTAGACCTGCACAAGCAATTGAATA	
R9-SpeF	ACATTATTACTAGTCGACGGCGGAAACGACACGAT	
R9-XbaR	AACCAACTCTAGAGGCTGAACAAGCAATTGAATA	
R12-SpeF	ACATTATTACTAGTTTAGGATTGGCTGAGGCAT	
R12-XbaR	AACCAACTCTAGAGCTTCAAGAACAATTGAAC	
R13-SpeF	AACAATACACTAGTAAAGCGACACAGAGCCCAACAA	
R13-XbeR	ACATTATCTAGACCTGCATAAACAAACAGATAC	
GA-M4-F0	GATGAGATAAACCAATACTAGTATTCCTGAACTTGTATTATTC	
GA-M4-R0	ATCAGCGGTACGCTTCATCTAGATTACTTAAACTAGAATCAA	
GA-M8-F0	GATGAGATAAACCAATACTAGTATCCTTTTACGTGTGCTGT	
GA-M8-R0	ATCAGCGGTACGCTTCATCTAGATTCCTTAAATCTGCAA	
GA-R2-F0	GATGAGATAAACCAATACTAGTGCTTGAAGGGGCAC	
GA-R2-R0	ATCAGCGGTACGCTTCATCTAGACCTGCACAAGCAAT	
GA-R9-F0	GATGAGATAAACCAATACTAGTCGACGGCGGAAACGACACGAT	
GA-R9-R0	ATCAGCGGTACGCTTCATCTAGAGGCTGAACAAGCAATTGAATA	
GA-R12-F0	GATGAGATAAACCAATACTAGTTTAGGATTGGCTGAGGCAT	
GA-R12-R0	ATCAGCGGTACGCTTCATCTAGAGCTTCAAGAACAATTGAAC	
GA-R13-F0	GATGAGATAAACCAATACTAGTAAAGCGACACAGAGCCCAA	
GA-R13-R0	ATCAGCGGTACGCTTCATCTAGACCTGCATAAACAAACAGAT	
GA-U6t-F	AGTTCCGATGAGATAAACCAATACT	
GA-Lb12-R	CTGCCATCAGCGGTACGCTTCATCT	
BAG-IDF	GGTCAACACTGTACACTCATCTCCT	T-DNA insertion identification
BAG-IDR	CCCCTGAGCTGGTACTGCTTCTTGC	
BADH1-F	GTGGAAAACCACTAGATGAAGCACT	Sanger sequencing
BADH1-R	CCAGCAGCTAGAGCAGGAGCAACTT	
BADH1-F2	ATGAAGCTGGTGCTCCTTTGTCATC	
BADH1-R2	CCCCAAATTGGATATTACACGATCTCAC	
BADH2-F	TGACTGTGGAAAACCGCTCGAT	
BADH2-R	GATGCCAACTCAGAGGGCTTCA	
BADH2-F2	GTCACGGTCGCAATTTTGTCAATCC	
BADH2-R2	CCCTTGTCAAGGTCAACATCCTCAA	
BADH3-F	TTTTCTGCATTCTTGCAACACAACATCTTA	
BADH3-R	GTCATCATCATTCCAATTTTGGTGCTTCCT	

E1-F	CTCAAAGCCCATCAAAGTTCACGAC	
E1-R	ACATAGCTCTTGGATGAAGACCATC	
Lox1-F	TCTTGGAAGGGATTAATACTTCGTTACC	
Lox1-R	CTGGACGAGCTAACTTTTCACTCTTATC	
Lox2-F	TGGAAGGGATTATTGTGTCGTTACC	
Lox2-R	CTGCTGGTGTCTCACTTGGAAC	
Lox3-F	GTCCTGTTCTTGGAGGAAATGACAC	
Lox3-R	GAAACTTAAGGGCCTGTTCACCATC	
BADH1-HTS-F	TGGTTGTTTTAACTACTATGCC	High-throughput sequencing
BADH1-HTS-R	AAATGAAGAAACAGCAAATACC	
BADH1-HTS-F2	CAAGTATTGTTACTTTGCTATGATT	
BADH1-HTS-R2	ACATAATTTGCAGATAATACTGCTT	
BADH2-HTS-F	GCAAGCAAAGAAACACCGATTA	
BADH2-HTS-R	CGGCTCCTTAAGAACATAACTC	
BADH2-HTS-F2	GCAATGCTAGTTTGACATTGTG	
BADH2-HTS-R2	CAAAACAAATGGAAACACGAAC	
BADH3-HTS-F	CTCTGACTTTATTATGTAGGATGAT	
BADH3-HTS-R	AAATGAAGAAACAACAAATACC	
E1-HTS-F	TTGGAAGATCAAGAAGACGCTAACC	
E1-HTS-R	TGTCCATGTCCCAAACTCTAAC	
Lox1-HTS-F	GTTTGACTCTTGAAGCCATTTC	
Lox1-HTS-R	CTTGAAGAACTTGCCATTAGAC	
Lox1-HTS-F2	GGGATTAATACTTCGTTACCAAC	
Lox1-HTS-R2	GGTTCCTTGGTTTGAAATGGCTT	
Lox2-HTS-F	GTTTGACTCTTGAAGACGTTCC	
Lox2-HTS-R	TATAAACTACTAGCTAAGTACG	
Lox2-HTS-F2	GGATTATTGTGTCGTTACCAAC	
Lox2-HTS-R2	TGGTTCCTTGGTTTGGAACGTC	
Lox3-HTS-F	CTAATAGTGAGAGTAGGAGCAA	
Lox3-HTS-R	TCATCAAAGCTATCAAACTCAC	
Lox3-HTS-F2	TCTTCCATTATTGCAATCTGCT	
Lox3-HTS-R2	ATCAGTTCGGAAGATTTCCTT	

Table S10. Sequences of target sites

Table S10. Sequences of target sites

**crRNA array**	**crRNA**	**gene**	**Target sequence**
a & b	BADH1&3	*BADH1*	**TTTC**CATAGGAAGGGATACAGGAGCAT
		BADH3	Same as the above
	BADH2	*BADH2*	**TTTC**AGCAAGGTCAGCATAGAACTCAA
	Lox1&2-1	*Lox1*	**TTTA**TAACACTAAACTTTACAAAAGCG
		Lox2	Same as the above
	Lox3-1	*Lox3*	**TTTA**GTCCATAAGTAAGAAAGTCAGAT
c & d	BADH1&2	*BADH1*	**TTTA**CTGGAAGCTCTGCAACTGGAAGC (for crRNA)
		BADH2	**TTTA**CTGGAAGCTCTGCAACTGGGAGC
	E1	*E1*	**TTTC**TTCACCAAATCTGCAGCCAGCAA
	Lox1&2-2	*Lox1*	**TTTA**TGTAAAACGCACCGGGGATTCCC
		Lox2	Same as the above
	Lox3-2	*Lox3*	**TTTG**ATGAAGTTCATGGACTCTATTCA

PAM sequences are highlighted in bold, and mismatches are indicated in red.

Sequences S1. Sequences of the six promoters and crRNA cassettes

GmScreamM4p (1.42-kb)

attcctgaacttgtattattcagtagatcgaataaattataaaaagataaaatcataaaataatattttatcctatcaatcatattaaagcaatgaatatgt
aaaattaatcttatctttattttaaaaaatcatataggtttagtattttttaaaaataaagataggattagttttactattcactgcttattacttttaaaaaaatc
ataaaggtttagtatttttttaaaataaatataggaatagttttactattcactgctttaatagaaaaatagtttaaaatttaagatagttttaatcccagcattt
gccacgtttgaacgtgagccgaaacgatgtcgttacattatcttaacctagctgaaacgatgtcgtcataatatcgccaaatgccaactggactacgt
cgaacccacaaatcccacaaagcgcgtgaaatcaaatcgctcaaaccacaaaaaagaacaacgcgtttgttacacgctcaatcccacgcgag
tagagcacagtaaccttcaaataagcgaatggggcataatcagaaatccgaaataaacctaggggcattatcggaaatgaaaagtAGCTCA
CTCAATATAAAAATCTAGGAACCCTAGTTTTCGTTATCACTCTGTGCTCCCTCGCTCTATTTCTCAGTCT
CTGTGTTTGCGGCTGAGGATTCCGAACGAGTGACCTTCTTCGTTTCTCGCAAAGgtaacagcctctgctcttgt
ctcttcgattcgatctatgcctgtctcttatttacgatgatgtttcttcggttatgtttttttatttatgctttatgctgttgatgttcggttgtttgtttcgctttgtttttgtgg
ttcagtttttttaggattcttttggtttttgaatcgattaatcggaagagattttcgagttatttggtgtgttggaggtgaatcttttttttgaggtcatagatctgttgt
atttgtgttataaacatgcgactttgtatgatttttttacgaggttatgatgttctggttgttttattatgaatctgttgagacagaaccatgatttttgttgatgttcg
tttacactattaaaggtttgttttaacaggattaaaagtttttttaagcatgttgaaggagtcttgtagatatgtaaccgtcgatagttttttttgtgggtttgttcac
atgttatcaaCcttaatcttttactatgtatgcgaccatatctggatccagcaaaggcgatttttttaattccttgtgaaacttttgtaatatgaagttgaaatttt
gttattggtaaactataaatgtgtgaagttggagtatacctttaccttcttatttggctttgtgatagtttaatttatatgtattttgagttctgacttgtatttctttga
attgattctagTTTAAGTAAT

The nucleotides in light blue indicate 5'-UTR, which is split by an intron.

GmScreamM8p (1.58-kb)

atccttttacgtgtgctgtgagacattatcatcaattgtgttgtatatgatatatagatatagatatataaatatatagattgagtgatataatatatttaaaat
ataaattatatatatgttttaatatatttttgcatatatatatatatttgtaaaaactagaagtattttttcatgagataattattatcgagttgaataagtctattat
ttgtgagagccaaccatatttatatatgtgattaaattttatctttgtgaaattaaaaataataaaaaataccttaaaatcataataatagaaaaacttata
tttataatttaccattatacttaaaaaaaaattaaataaatattataaatataaatactatcgagtaatggccgcgctagggtttttgagaaaaaatcttccc
acgcactcaactgcactgtacggcgtcgttttcacagccgcataatagaagccgcgttccccaacccttcctcacaacattctcggaccctccagca
ccgtcacccaaacaaatatccacgcggtagtaggcgcgtgaaacaaactctaatccgaactacgagacgtgagaagcacgcgctttagcgagc
gtttcaattgtcgctacgaaagcagagaaggatacaaacggaactagggtaaattagtaagggtaatttcgtaaacagaagaaaagagttgtagc
tataaataaaccctctaaccctcgtcgcattACTTCTCTTCACACCTTTGTTCACTCTTCTTCTCTTGCGGCTAGGGT
TTTAGCGCAGCTTCTTCTAGgttcgttatctaccaccgttctatggattttattccttctattcgtgtttattctattggtttatgttgcttgcaatatgt
tttttctgaatctgtcgtcgttgtcttcaattttatccatgtttcagagatcaattttgtttgtgtagtatgtgcttattcttcttcttttcgttcgagttgttaataacggt
gctatggtgttttcaaaagtgtttttttttattactttttgatttaaagttttttttggtaaggcttttatttgcttgttatattcaaatctttggatccagatcttatataagttt
ttggttcaagaaagtttttggttactgatgaatagatctattaactgttactttaatcgattcaagctaaagtttttttggttactgatgaatagatctattatctgtt
acttttaatcggttcaagctcaagtttttttggttactgatgaatagatctatatacgtcacagtgtgctaaacatgcccttgttttatctcgatcttatgtatggg
agtgccataaattttgttatgtctattttttttatctgttggaatcatactgagtttgatgcgttacgattgagcatacctatttttgggcttgttgtatggtgggtattt
agatcttaatctttttatgcttatgaaaggttttgtaatgacaaaggtcttaatgttgttaaacttttatttttactttatatggtgtgttgatgtgttatggttttgaca
acttttttttttttctggatttttgcagATTTAAGGAA

The nucleotides in light blue indicate 5'-UTR, which is split by an intron.

GmRPS5-2p (1.22-kb)

gcttgaaggggcacccacattttctagtccctgtgtatcttctgataaattcttttttcctacacttatactcgctactcctttcacagccaattaacacaaac
gtagtccttcctctactacccgtgtttgtatctgaccttaaaatcaccgccacaaatccgttttcatgagcaacggatcgagcccactgcaaaacatcct
ctcgacactcaaacacctacaaagcaatccacataatttaagttttctaccagtattcattttattaaatctgtgacaaacattaacattataacctgaga
agtatttaacgcatccgaacaatcaacatgcggttcgttcacaccacatgcttcttcattttcataatccatattctcttgttcagacattatttcatacatcc

actcatcttcgtccatctaaccaacccaaacaaaaaatgaccatacaaacaaattaataatttaaaaaaaatggaaactaaattcaaaaacatc
aaattttaaaaatagttactgttacagatcaagttgatccgtaagtgacttacggatcaatttgatctgtaagttggtgtgttacagttacggatcaacttg
atccgtaagtgatccgtacgttcgtgacatagccaccaccttctgcgtacctcgtttgtcactgccgaccaccgcaaaacaccttcaccttcaccgca
cctaactgttcacaacaaaccaacgaaccggagacaatggcgcacgaaaccacaaaaaacagaaaaacaatgaacaaaaatggcagct
gtgtgaagcgcaggaaaaaaaaggttttttaatgaaaaaaaaacaccagggacatttttaccatttataaattttgctagtacaccagcaaaaatg
ctgggtgtacctagcagcactcaaatgaaaaccaccaagggcccaagtcctcggacatatcctatagaacactagacaaacaatgggccgggc
ctaacaatttaccttagggttttctcgatcattataaatacccaaatgtccAAGCGAAAAACCTAGGGTTTTTGTCGTCGCTACT
AAGAGTTGCTGCTTCTTCAGCCACTCCGCACCTCGCAGCCTCTGTGCCCTCAACAAgttcgttgtcttccatttt
cttcttcttgagttcttctctttctctctcttcccttcgctaacactctctctattcaattgcttgtgcagGTCTAGA

The nucleotides in light blue indicate 5’-UTR, which is split by an intron.

GmRPS5-9p (1.31-kb)

tcgacggcggaaacgacacgatggtggtggcgacgtccttaaagtcgacaatggaagcaacgaacgacggtggcacaacacaaactaaaga
agacgatggagggaggcaaaggaagaagatcatgtgcagcgcatacaaacaaagaagaggagggaaggtttttaacattcaagtgaaggac
atttttgtcacttcacttaaattgctgtgtgcacaaacaatattactgggtgcacctaacaacggctgaaatacaaataagagaatgcaaaatttaggg
tattgctatgtacacctagtgaaatcccaacaatttcatgatttttctattttgccctttcgtaaaagggatacggattcatatgaggcttacggattagttga
tccatatgagcctaatccgtaagccttacagattggactcatatggatcagttgatccgtaagtgtttttcaattttttattttttttcatacaaatcagccgca
agtgttttattaaaattttttttcaggttattagcacaagactttaaccaactgagctaataggtcaattatattataaaataaataatgttgttatatataaca
gtaaaatttctaatgtatatttaatgcacatgtaaaattaaataataaattttatgataattaattttgatataactcatactaattatttaatctgtatattttttat
aaataaaataatatttaatattaaaaaaattaatttattaataaattaaaaaaacatttttgaattttttttaaaaaaacacttatgaattacgtaatccgtat
gactcatacggattacgtaatctgttcataatccatatgaattaatccgtatgactcatacggatcacataatttgtgtgaggtagacggattatgtaatc
cttaaaaataaaaaaacacttaagggcagtttcggaaattttgtttaatgctgggtgcacaagcaatatgtctggtgcacctagcaaaaccccaaaa
tttaaatgcctaaaatgaaaaccaccaagggcccggggtccaaagacacacaaaccaataacatgggccgggcctaaccattaggGTTTT
TTCCATTGCTATAAATACCCAAATGTCATAGCGAAAGAACATAGGGTTTTTGTCGTTGCGACTAAGAGT
TGCTGCTTCTTCCGCCACTCAGCACTTCGCTCCCTCTGTGCTTCACCAAgttcgttctcttccattttcttctgcttctgtt
cttctctttctctctcttcccttcgctaactcgttctctatattcaattgcttgttcagCC

The nucleotides in light blue indicate 5’-UTR, which is split by an intron.

GmRPS5-12 (1.57-kb)

tttaggattggctgaggcatgcaaaatgcagagctgaagtaattttactgtcacaagtaataggtaatatattctctatgaataggatggcaataacaa
aagagcattagcttctgtgaaccactcaccttaagcgaagccatgaagtgagttcaagaagtgatgctttttcatatcccaatgaagcccccctaa
ataatgagtactgagatagattgaaaaaataatttaaaaaaaatagaatggaatgcgcgccaggtaggggtcgaacctacgactttctgcttagga
aacagacgctctatccactgagctacaagcgctgaaagttaataaagtttttatgaaaaattatacatatcctaattacgaactttaccacagatcaat
tccatctgacatatgatgtgtatgtaagcgaaggaaacatgaatagacgatatcagaattttcatgtattgtgattgtgattaaggttcctttagaataata
tgtgaaatttaaaaataaaataaaattctgacacaaattaggatgttcattttagattttttttcctttttatatattttttttcatatggattgatctttttcttttcgac
aaacttaccatggttttgtgtgagtgtgtgaatttacttgaaaaccttttctattcatttactaaggttgagtgttgatttgaaaaaaaaaaaaagaattaatta
agtttttatttttttaaataaaagtttttagttccttatttttaatttttttaagttcctattttttttattaattttttatcagcattttaagttcctttgataattttagtctcatttt
atttttattactcaaaatgtgtttgtttatttattttgagtctctcatttattttatatttaagattaattatgggtaatcaaaataaatgagtgactaaaactaaaa
aaataaaaaaatattaaaaatgttaacaaaatattaacgaagaattaaaaattgtgaaaaaaatttaagaaactaaaaattagaattttaaaaaatt
tagagacaaaaaattaattaatcctaaaatatatacaaaataaaaaattgaactaaagccataaaatatttaaaatacaagaattaaaatcaaatg
aaaaaaaaataaagaaactaaaatgacgatgatgaagtatgtcaggacgaaaattaaaattttttcaaaattttaaaatagtttaataatggtcttta
ggttttagctttgtttatgccactctgagggtataaatttgagcaactagggtTTTAAGTTGTGGTTCTTGTTTGCGAGATAGAAG
CGTAAGCAGCGGCTACCCTAACCTCGTCTTCCTCTCTACTAATTTCAGGTACAATCACTGCCATTTCA
ATCTTTTCATTGAAAGTCTTGTTCTTTCTGCCGCTCCACCATTCGTTAGATATCTCTCTTAATCATCGAA

GGGTTTCAACACTCTGCTTCGCTCTGCATTTGTTCCGTTCCGTTCCTCTTTTTTCTTTTTTAATTTTTTT
ATTCTCGCTTTCTGTAATTGTATCTGTTCAATTGTTCTTGAAGCT
The nucleotides in light blue indicate 5’-UTR.

GmRPS5-13 (1.48-kb)

aagatgattgtcgtgtgatgtattgcatttattatttgtcatttcttttattaatttgtataaaataaatagattaagtaaaatatatacaattttgtactaaaaaa
attatgtcatgcttataagatataatatatatcatgataatcataacttacatctccattttcaatttttactctttatatctaaattttataatatattttatgtttcatt
tattttatatttgagactaattgtgaacaataaaaataaaggatttggctaaaaatggacaattttttaaacaaactaaaaatgttgacaagaaattaa
agaaagattaaaggttgtaaaaaaaatagaaaaattaaaaatctatatatctatatattatctacctctctctctttatatatatatatatatatatata
tatatatcatttaaaataggattattgactatatttgtttgcatatgtgacaaatggtgattaaattaagtctcactaattaatttattttttccattaaaacaac
aaataaaataaagttcaatcaaaacatgaaaaagaaagaaaataattaatttatttggaacctttgtttaaagttattatgttatattttagaaatcacat
tatgagaaatttatatatctatatctatattatttaaaacaagattgttgattgtacctgatcttgcataattagttttttttttgttaaaacaaataaaataaattt
caaccaaaacatgaaaaaaaagaattaatttatttaggacgtttgattataggtatcatattatatttttggaaataaaactttgaagaatattaatctttta
taatattttttctcatttaatatatttttaaaaaattaatattaataaaaaactcatttactttaaaaaaagttggtataatttatttgcttaacgctaaaatgattg
atttaaataattgattaaaataattaatatatttattttgataatcaagataattatagatttgaagattcaattttttaaactatgaaaatgttgaaatataga
aactaaaataaaattaaaaaaaaaagaaaccaaagagaaataatgaaacatgagaataccaaaattttaaaataGCTTAATGCTGTT
TTAGGTTTTAGCTTGGTTTATGCCATTCCGATATAAATTTGAGCAACTAGGGTTTTTACTTGTCCTTCTT
TGCGAGATTGAATCGTAAGCAGCGGCTACCCTAATCCTCTCCTCTTCCTCTCTACTAATTTCAGgtacact
cactcactggcatttcaatttttcattgaaagtgttgttctttctttcgctgcaccctccaacacttcattcgttagatatctctcttaattatcgaaacggtttca
acactctgcttcgttctgcattggttctcttcctttcctcttttttccctttctgtaattgtatctgtttgtttatgcagGTCTAGA
The nucleotides in light blue indicate 5’-UTR, which is split by an intron.

Sequence of pAGC-GmU6 (partial)

*Bsa*I-*Bbs*I-*Bbs*I-HDV-pT-GmU6p-DR-*Bbs*I-*Bbs*I-*Bsa*I (pT, U6_29t)
TTCGCTACCTTAGGACCGTTATAGTTACGGTCTCAAGATATGTCTTCACAACCAATGAAGACAGTTG
GCCGGCATGGTCCCAGCCTCCTCGCTGGCGCCGGCTGGGCAACATGCTTCGGCATGGCGAATGG
GACTTTTTTTTGGATAGAATTTCCCAGCTTTTTTGCGTGTTTCAGCTCTCATGATCCAAAATAAATGGTA
AAATGTCAAATCAAAACTAGGCTGCAGTATGCAGAGCAGAGTCATGATGATACTACTTACTACACCGAT
TCTTGTGTGCAGAAAAATATGTTAAAATAATTGAATCTTTCTCTAGCCAAATTTGACAACAATGTACACC
GTTCATATTGAGAGACGATGCTTCTTGTTTGCTTTCGGTGGAAGCTGCATATACTCAACATTACTCCTT
CAGCGAGTTTTCCAACTGAGTCCCACATTGCCCAGACCTAACACGGTATTCTTGTTTATAATGAAATG
TGCCACCACATGGATGTAATTTCTACTAAGTGTAGAATGTCTTCATGGTGGTGTGAAGACATGCCGT
GAGACCAATAGGGATAACAGGGTAATGTATGGT
(Two extra T bases in S2 do not affect editing efficiency)

Sequence of pGmU6-4x (partial)

*Bsa*I-S1-DR-S2-HDV-pT-GmU6p-DR-S3-DR-S4-*Bsa*I (pT, U6_29t)
TTCGCTACCTTAGGACCGTTATAGTTACGGTCTCA**AGAT**NNNNNNNNNNNNNNNNNNNNNNNTAAT
TTCTACTAAGTGTAGATNNNNNNNNNNNNNNNNNNNNNNNTTGGCCGGCATGGTCCCAGCCTCCT
CGCTGGCGCCGGCTGGGCAACATGCTTCGGCATGGCGAATGGGACTTTTTTTTGGATAGAATTTCC
CAGCTTTTTTGCGTGTTTCAGCTCTCATGATCCAAAATAAATGGTAAAATGTCAAATCAAAACTAGGCT
GCAGTATGCAGAGCAGAGTCATGATGATACTACTTACTACACCGATTCTTGTGTGCAGAAAAATATGTT
AAAATAATTGAATCTTTCTCTAGCCAAATTTGACAACAATGTACACCGTTCATATTGAGAGACGATGCTT

CTTGTTTGCTTTCGGTGGAAGCTGCATATACTCAACATTACTCCTTCAGCGAGTTTTCCAACTGAGTC
CCACATTGCCCAGACCTAACACGGTATTCTTGTTTATAATGAAATGTGCCACCACATGGATGTAATTTC
TACTAAGTGTAGATNNNNNNNNNNNNNNNNNNNNNNNTAATTTCTACTAAGTGTAGATNNNNNNNN
NNNNNNNNNNNNNNNGGCCGTGAGACCAATAGGGATAACAGGGTAATGTATGGT

(Two extra T bases in S2 do not affect editing efficiency)

Primers

oS1-F	**AGAT**NNNNNNNNNNNNNNNNNNNNNNNTAAT	5'-Phosphorylation
oS1-R	**AGAA**ATTANNNNNNNNNNNNNNNNNNNNNNN	5'-Phosphorylation
oS2-F	**TTCT**ACTAAGTGTAGATNNNNNNNNNNNNNNNNNNNNNNN	5'-Phosphorylation
oS2-R	**CCAA**NNNNNNNNNNNNNNNNNNNNNNNATCTACACTTAGT	5'-Phosphorylation
oS3-F	**TAGA**TNNNNNNNNNNNNNNNNNNNNNNNTAATTTCTACT	5'-Phosphorylation
oS3-R	**ACTT**AGTAGAAATTANNNNNNNNNNNNNNNNNNNNNNNA	5'-Phosphorylation
oS4-F	**AAGT**GTAGATNNNNNNNNNNNNNNNNNNNNNNNG	5'-Phosphorylation
oS4-R	**CGGC**CNNNNNNNNNNNNNNNNNNNNNNNATCTAC	5'-Phosphorylation

Generation of short inserts by annealing two 5'-phosphorylated oligos (primers)

oS1-F + R = Ins-S1

oS2-F + R = Ins-S2

oS3-F + R = Ins-S3

oS4-F + R = Ins-S4

***Bbs*I-based Golden Gate Cloning**

pAGC-GmU6 + Ins-S1 + Ins-S2 + Ins-S3 + Ins-S4 = **pGmU6-4x**

Colony PCR primers: oS1-F + oS4-R = 574-bp

Sequencing primers: M13F and M13R

4xcrRNAs (AtU6-2x + GmU6-2x)

AtU6-26p-DR-S1-DR-S2-HDV-U6-29t-GmU6p-DR-S3-DR-S4-HDV-U6-26t

CGACTTGCCTTCCGCACAATACATCATTTCTTCTTAGCTTTTTTTCTTCTTCTTCGTTCATACAGTTTT
TTTTTGTTTATCAGCTTACATTTTCTTGAACCGTAGCTTTCGTTTTCTTCTTTTTAACTTTCCATTCGGA
GTTTTTGTATCTTGTTTCATAGTTTGTCCCAGGATTAGAATGATTAGGCATCGAACCTTCAAGAATTT
GATTGAATAAAACATCTTCATTCTTAAGATATGAAGATAATCTTCAAAAGGCCCCTGGGAATCTGAAA
GAAGAGAAGCAGGCCCATTTATATGGGAAAGAACAATAGTATTTCTTATATAGGCCCATTTAAGTTG
AAAACAATCTTCAAAAGTCCCACATCGCTTAGATAAGAAAACGAAGCTGAGTTTATATACAGCTAGA
GTCGAAGTAGTGATTGTAATTTCTACTAAGTGTAGATNNNNNNNNNNNNNNNNNNNNNNNTAATTTC
TACTAAGTGTAGATNNNNNNNNNNNNNNNNNNNNNNNTTGGCCGGCATGGTCCCAGCCTCCTCGC
TGGCGCCGGCTGGGCAACATGCTTCGGCATGGCGAATGGGACTTTTTTTTGGATAGAATTTCCCAG
CTTTTTTGCGTGTTTCAGCTCTCATGATCCAAAATAAATGGTAAAATGTCAAATCAAAACTAGGCTGCA
GTATGCAGAGCAGAGTCATGATGATACTACTTACTACACCGATTCTTGTGTGCAGAAAAATATGTTAAA
ATAATTGAATCTTTCTCTAGCCAAATTTGACAACAATGTACACCGTTCATATTGAGAGACGATGCTTCTT
GTTTGCTTTCGGTGGAAGCTGCATATACTCAACATTACTCCTTCAGCGAGTTTTCCAACTGAGTCCCA
CATTGCCCAGACCTAACACGGTATTCTTGTTTATAATGAAATGTGCCACCACATGGATGTAATTTCTAC
TAAGTGTAGATNNNNNNNNNNNNNNNNNNNNNNNTAATTTCTACTAAGTGTAGATNNNNNNNNNNN
NNNNNNNNNNNNGGCCGGCATGGTCCCAGCCTCCTCGCTGGCGCCGGCTGGGCAACATGCTTCG
GCATGGCGAATGGGACTTTTTTTTGCAAAATTTTCCAGATCGATTTCTTCTTCCTCTGTTCTTCGGCG
TTCAATTTCTGGGGTTTTCTCTTCGTTTTCTGTAACTGAAACCTAAAATTTGACCTAAAAAAAATCTCA

AATAATATGATTCAGTGGTTTTGTACTTTTCAGTTAGTTGAGTTTTGCAGTTCCGATGAGATAAACCA ATA

(Two extra T bases in S2 do not affect editing efficiency)

***Bsa*I-based Golden Gate Cloning**

pBG-M4/M8/R2/R9/R12/R13Lb + pGmU6-4x(a–d) = pM4a–d/pM8a/pR2a/pR9a/pR12a/pR13a

Colony PCR and sequencing primers

U6-26p-F	TGTCCCAGGATTAGAATGATTAGGC
U6-26t-R	CCCCAGAAATTGAACGCCGAAGAAC
U6-26p-F + U6-26t-R = 1.0-kb	

Methods S1. Vector construction, mutation analysis, and related procedures

Vector construction

All primers used in this study are listed in Table S9, sequences of all target sites are in Table S10. The six promoter sequences, the cloning cassettes for four-crRNA assembly, and the final expression cassettes are compiled in Sequences S1.

To isolate GmScreamM4 free of its internal *Hin*dIII site, we performed a two-step PCR on *Glycine max* (cv. Tianlong #1) genomic DNA. First, we generated two overlapping fragments with M4-SpeF/M4-mHiR and M4-mHiF/M4-XbaR. Using the mixed first-round products as templates, we then amplified the full-length promoter with M4-SpeF/M4-XbaR, purified the amplicon, and ligated it into a blunt-end vector to yield pABC-M4p. For GmScreamM8 and GmRPS5-2/9/12/13 (*Glyma.02G167900*, *Glyma.09g103400*, *Glyma.12g201900*, and *Glyma.13g300300*), we amplified the corresponding fragments with M8-SpeF/-XbR, R2-SpeF/-XbR, R9-SpeF/-XbR, R12-SpeF/-XbR, or R13-SpeF/-XbR, purified the products, and cloned them into the same blunt-end vector to obtain pABC-M8p, pABC-R2p, pABC-R9p, pABC-R12p, and pABC-R13p (where M8 denotes GmScreamM8 and R2/9/12/13 denote GmRPS5-2/9/12/13). We replaced the AtRPS5a promoter in pBG-ttLbUV2 with each of the six promoters via Gibson Assembly. Specifically, using GA-U6t-F, GA-XXX-F0, GA-XXX-R0, and GA-Lb12-R (XXX = M4, M8, R2, R9, R12, or R13), we amplified each promoter from its pABC source, purified the fragments, and assembled them with *Xba*I/*Spe*I-linearized pBG-ttLbUV2, yielding pBG-M4Lb, pBG-M8Lb, pBG-R2Lb, pBG-R9Lb, pBG-R12Lb, and pBG-R13b.

We inserted a synthetic fragment into a pUC57-derived backbone to generate pAGC-GmU6Lb. To build a four-crRNA array, we performed *Bbs*I-based Golden Gate reactions with pAGC-GmU6Lb and four short inserts (each produced by annealing a pair of 5′-phosphorylated oligonucleotides), yielding pGmU6-4x and subsequently pGmU6-4x a–d (Table S10; Sequences S1). Next, *Bsa*I-based Golden Gate assembly between one of pGmU6-4x a–d and one of pBG-XXXLb (XXX = M4, M8, R2, R9, R12, or R13) produced

pM4a, pM8a, pR2a, pR9a, pR12a, pR13a, pM4b, pM4c, and pM4d (Sequences S1).

Generation of transgenic plants and mutation analysis

We introduced each of the nine vectors into *Agrobacterium tumefaciens* EHA105 and transformed soybean (*Glycine max*, cv. Tianlong #1) cotyledonary nodes under glufosinate selection. We verified transgenic lines by PCR with BAG-IDF/BAG-IDR.

For editing assessment, we amplified genomic regions spanning each target with primers in Table S9 and performed high-throughput sequencing (HTS) (~10,000 reads per amplicon). We analyzed data using the Hi-TOM 2.0 workflow (Sun et al., 2024) with a 1% calling threshold. We quantified editing using two complementary metrics. In the reads-based metric, for each line we calculated the fraction of reads carrying edits at a given target; the mean across lines with the same vector defines that target's editing efficiency. In the sorting-based metric, mutation efficiency equals the proportion of transgenic plants with detectable edits. Genotypes were assigned as follows: homozygous (Ho) if ≥95% of reads supported the same mutation; biallelic (Bi) or multiallelic if ≥95% of reads comprised more than one mutation type; heterozygous (He) with or without additional mutations if ≥45% but <95% of reads carried mutations; and chimeric (Chi) if <45% of reads carried mutations.

Identification of T-DNA-free T1 mutants and quantification of 2-AP content

To assess whether the mutations in the six genes in T0 plants were heritable, T1 seeds were harvested from eight surviving T0 plants, grown in soil, and their genotypes analyzed by HTS. We identified T-DNA-free plants using PCR with primers BAG-IDF/BAG-IDR (Table S9). T1 plants lacking the expected amplicon were classified as T-DNA-free.

The 2-AP content was determined by gas chromatography–mass spectrometry (GC–MS). All plants used for analysis were grown in a controlled growth chamber under a 14/10 h light/dark cycle at 28/25°C. Fresh leaves were harvested from four-week-old plants. The harvested leaves were immediately flash-frozen in liquid nitrogen and ground into a fine powder. Subsequently, 0.1 g aliquots of the powdered samples were extracted with 1 mL

anhydrous ethanol. The mixtures were subjected to ultrasonic extraction for 30 min, followed by static extraction at room temperature for 2 h. After centrifugation at 12,000 rpm for 10 min at 4°C, a 1 mL aliquot of the supernatant was filtered through a 0.22 μm membrane filter. Finally, the processed samples were analyzed for 2-AP using GC–MS.

References

Sun, T., Liu, Q., Chen, X., Hu, F. and Wang, K. (2024) Hi-TOM 2.0: an improved platform for high-throughput mutation detection. *Science China Life Sciences* **67**, 1532-1534.
